# Establishment of Efficient Somatic Embryo Maturation System of *Pinus elliottii*

**DOI:** 10.3390/plants14131985

**Published:** 2025-06-29

**Authors:** Lin Xu, Zhaolei Deng, Shan Hu, Qian Liu, Qifu Luan, Chunxia Yang

**Affiliations:** 1 Jiangxi Academy of Forestry, Nanchang 330013, China; xulin@njfu.edu.cn (L.X.); dengzhaolei97@126.com (Z.D.); lkyhushan@163.com (S.H.); lq_lky18670223641@126.com (Q.L.); 2 Jiangxi Provincial Key Laboratory of Improved Variety Breeding and Efficient Utilization of Native Tree Species, Nanchang 330013, China; 3 Research Institute of Subtropical Forestry, Chinese Academy of Forestry, Hangzhou 311400, China; qifu.luan@caf.ac.cn

**Keywords:** *Pinus elliottii*, somatic embryogenesis, somatic embryo maturation, hormone ratio, activated carbon

## Abstract

*Pinus elliottii*, a key economic conifer in southern China, requires efficient propagation methods to meet demand for elite germplasm in resin and timber production. While somatic embryogenesis-based plant regeneration has been successfully achieved in *Pinus elliottii*, large-scale production remains challenging. Our results demonstrate that the genotype of *Pinus elliottii* significantly influences the induction rate of embryogenic callus. During somatic embryo maturation, the liquid–solid induction method increased the number of mature embryos by 25.85 times. Maturation efficiency was further enhanced by a 3-week pretreatment followed by the application of 9 mg/L ABA, 0.5 mg/L PSK, and 6 mg/L COS. Additionally, the incorporation of activated carbon significantly promoted both the maturation and germination of somatic embryos. In the somatic embryo maturation stage, 1 g/L activated carbon induced 288.67 mature embryos per gram of embryogenic callus, resulting in a total of 1452 embryos. During germination, the application of 4 g/L activated carbon achieved a germination rate of 63%, and the survival rate of somatic embryo-derived seedlings reached 85%. This study not only identifies the optimal conditions for somatic embryogenesis in *Pinus elliottii* but also establishes an efficient protocol for somatic embryo maturation induction, providing a crucial scientific foundation for the rapid propagation and seedling production of *Pinus elliottii*.

## 1. Introduction

*Pinus elliottii*, commonly known as Slash Pine, is an evergreen tree species native to the southeastern United States. Since its introduction to China, it has been extensively planted in the Yangtze River Basin and southern China [1]. The species is particularly valued for its high yield and exceptional resin quality, making it a key source of both resin and timber in southern China [2]. Beyond its timber and resin production, the pine needles of *Pinus elliottii* are utilized in traditional medicine, and its pollen is considered a medicinal food. Moreover, beneath its canopy, valuable Chinese medicinal herbs and edible fungi can be cultivated, further enhancing its economic value. Thus, *Pinus elliottii* plays a vital role in afforestation efforts, as well as in timber and resin production, making in-depth research into this species of practical significance for improving forestry income. Currently, there is an increasing market demand for improved varieties of *Pinus elliottii*. However, the existing improved varieties are limited, the breeding cycle is lengthy, and there is a shortage of young seedlings. To address these challenges, somatic embryogenesis has emerged as a promising solution. Somatic embryogenesis is a process in which somatic cells generate embryos through a pathway similar to zygotic embryogenesis, triggered by specific hormones or stress conditions [3]. This process not only serves as a valuable biological model for studying early zygotic embryogenesis in higher plants but also holds broad application prospects and significant economic value in large-scale propagation, germplasm preservation, and genetic transformation [4]. Although somatic embryogenesis-based seedling systems have seen significant advancements for many conifers, their widespread application in practical reproduction remains limited due to their suitability for only a few genotypes [4]. Consequently, addressing the challenges related to genotype limitation and the low efficiency of somatic embryo maturation is crucial for the broader adoption of somatic cell breeding technologies [5]. However, for some conifer species, including pine and common juniper, somatic embryogenesis can only be induced using zygotic embryos at the polyembryonic division stage [6,7,8]. This stage requires a precise collection window, which poses practical difficulties. Furthermore, the frequency of embryogenic callus induction in conifers is relatively low and often varies depending on the genotype of the mother tree [9,10].

During the maturation of somatic embryos in conifers, the proliferation of early somatic embryos ceases, and the accumulation of essential nutrients such as starch, proteins, and lipids begins [4]. Subsequently, the embryos progress through distinct developmental stages: globular, heart, torpedo, early cotyledon, and cotyledon stages. A significant amount of research has been dedicated to enhancing embryo initiation or maturation by modifying the composition of the culture medium. This includes adjustments to plant growth regulators, polyamines, and hormone inhibitors [11,12,13,14]. For example, exogenous plant hormone Brassinolide (BL) has also been shown to enhance somatic embryogenesis in *Pinus koraiensis* by modulating endogenous hormone levels, promoting antioxidant enzyme activity, and facilitating metabolic processes [15]. The maturation of spruce somatic embryos, for instance, is heavily influenced by the presence of abscisic acid (ABA) and osmotic stress from carbohydrates in the medium [16,17]. According to Varis et al., the optimal ABA concentration for somatic embryo maturation in *P. abies* was found to be 30 μM [18]. Their study also demonstrated that high molecular weight compounds, such as polyethylene glycol (PEG 4000), positively influenced the quality of somatic embryos and increased the number of mature spruce embryos [19,20]. Similarly, the development of mature somatic embryos in *Pinus sylvestris* required high concentrations of abscisic acid (80–90 μM) as well as the addition of penetrants, such as 9–10 g/L of gellan gum in the medium [21,22].

For the somatic embryogenesis of *Pinus radiata* and *Pinus halepensis*, changes in the chemical environment during the maturation stage—particularly the amino acid composition, carbon source, and their concentration—have a decisive impact on the maturation process of pine trees and significantly improve the success rate of somatic embryogenesis [23]. Furthermore, the synchronization of somatic embryogenesis in *Pinus elliottii* × *Pinus caribaea* can be effectively enhanced by establishing a liquid culture system, combined with the synergistic effects of ABA, ammonia (NH_4_^+^), nitrate (NO_3_^−^), low temperature, and PEG, providing a stable and efficient technique for large-scale seedling propagation [24]. Finally, variations in temperature and plant hormones also influence the stress resistance as well as the growth and development of embryos during somatic embryogenesis in *Pinus radiata*. Notably, high-temperature treatment delays drought resistance but promotes plant growth under optimal water conditions. Hormones regulate stress responses during the early stages of embryogenesis and have long-term effects on subsequent developmental stages [25]. The low efficiency of somatic embryo maturation in *Pinus elliottii* remains a major technical bottleneck limiting its commercial application [26]. Jain et al. studied immature zygotic embryos from four different genotypes of *Pinus elliottii* and successfully generated early somatic embryos using media containing varying proportions of auxin and cytokinin. They suggested that both genotype and medium composition were key factors influencing the production of embryogenic callus during the development of immature zygotic embryos [27]. The addition of 2,4-dichlorophenoxyacetic acid (2,4-D, 6 mg/L), 6-benzylaminopurine (BA, 1 mg/L), and kinetin (2 mg/L) significantly enhanced the callus induction efficiency in slash pine, reaching up to 15.67 ± 1.89% [28]. Incorporating glutamine into the somatic embryo maturation induction medium also resulted in an increase in somatic embryo production [28]. Newton et al. induced embryonic stalk cell mass from immature zygotic embryos of *Pinus elliottii* on LP medium supplemented with 1 mg/L 2,4-D and 0.5 mg/L BA. These embryonic stalk cell masses were transferred to fresh medium every three weeks, eventually leading to the regeneration of plants [29]. Hu et al. optimized the conditions for somatic embryo maturation induction in *Pinus elliottii*, achieving an average of 23.3 cotyledonary embryos per gram of medium. The germination rate was 32.1%, and the transplant survival rate was 47.8% [30]. Yang et al. have optimized the concentrations of ABA, PSK (Phytosulfokine), maltose, inositol, and other substances in the induction of somatic embryos, increasing somatic embryogenesis efficiency to 36% [26]. Additionally, several researchers have shown that family had a significant effect on embryogenic callus induction. Genotypic differences notably influenced the somatic embryo yield in resistant *Pinus elliottii*, with the quantity of embryogenic callus declining with an increasing number of subculture cycles [26].

This study aimed to establish an effective somatic embryogenesis system to overcome the low maturation efficiency of *Pinus elliottii* somatic embryos commonly encountered in practical production. To achieve this, immature zygotic embryos from 10 open-pollinated *Pinus elliottii* families were selected for investigation. This study assessed the impact of maternal tree genotype on embryogenic callus induction, the influence of exogenous phytohormones and carbon sources on callus proliferation, and the impact of ABA, PSK, COS (Chitooligosaccharide), and AC (Activated Charcoal) on somatic embryo maturation. The optimized somatic embryogenesis system established in this study provides a reliable platform for germplasm innovation and genetic improvement, while also offering essential technical support for the commercial production and genetic enhancement of *Pinus elliottii* seedlings.

## 2. Results

### 2.1. Effect of Genotype on Callus Induction of Pinus elliottii

**Figure 1:**
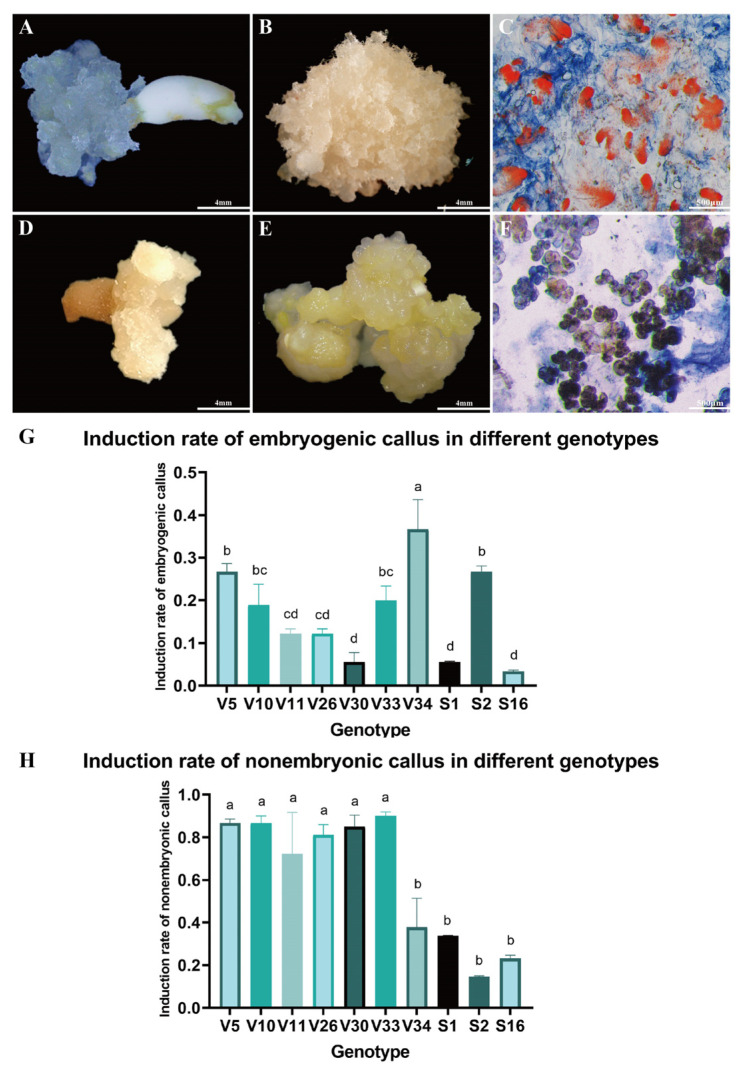
Callus Induction in *Pinus elliottii*. (**A**) Immature embryos inducing EC. (**B**) Embryogenic callus. (**C**) Acetocarmine-Evans blue staining of EC observed under electron microscopy. (**D**) Immature embryos inducing NEC. (**E**) Non-embryogenic callus. (**F**) Acetocarmine-Evans blue staining of NEC observed under electron microscopy. (**G**) Induction percentage of EC across different genotypes. (**H**) Induction percentage of NEC across different genotypes. Letters indicate significant differences among various treatments. *p* < 0.05. Bar: SE error bars.

Immature embryos of *Pinus elliottii* were cultured on induction medium to stimulate callus formation (Figure 1A,D). After six weeks, substantial callus proliferation was observed on the explants. Based on distinct morphological characteristics, the calli were classified into two types. One type, characterized by a rice-white, compact structure with transparent, filamentous protrusions on the surface. This callus demonstrated the capacity to form somatic embryos and was identified as embryogenic callus (EC) (Figure 1B). The other type presented a light yellow, loose, granular morphology with a smooth surface and lacked embryogenic potential, thus being categorized as non-embryogenic callus (NEC) (Figure 1E).

The formation of EC, NEC, or both concurrently was influenced by multiple factors, including genotype. Acetocarmine-Evans blue staining revealed significantly higher cell viability in EC compared to NEC. Microscopic examination showed that EC, characterized by well-defined embryonal heads and densely aggregated embryonal cells, stained bright red (Figure 1C). In contrast, NEC exhibited a loose structure with lower cell viability and lacked the embryonic stalk (Figure 1F). These structural differences in tissue and cells will be further analyzed in subsequent studies through statistical data and result evaluation.

Further examination of callus induction across ten genotypes revealed that the EC induction rate of the V34 genotype was significantly higher than that of the V5 and S2 genotypes, and markedly superior to other genotypes (*p* < 0.05) (Figure 1G). Conversely, the NEC induction rate for V34, S2, and S16 genotypes was significantly lower compared to the other seven genotypes (*p* < 0.05) (Figure 1H). These findings suggest that genotype plays a significant role in influencing the induction rate of EC in *Pinus elliottii*, with the V34 genotype exhibiting a notably higher induction rate (*p* < 0.05).

### 2.2. Suspension Culture of Embryogenic Callus of Pinus elliottii

**Figure 2:**
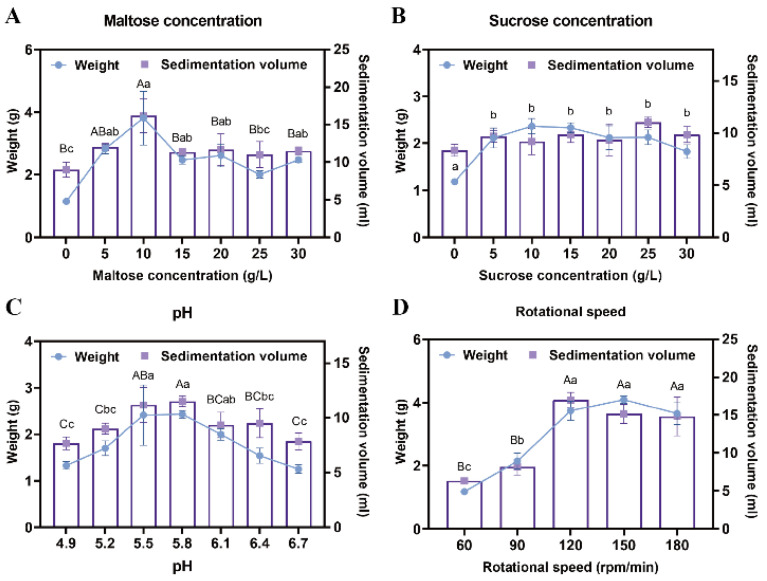
Screening of suspension culture conditions for *Pinus elliottii* embryogenic callus. (**A**) The effect of maltose concentration on the proliferation of EC. (**B**) The effect of sucrose concentration on the proliferation of EC. (**C**) The effect of pH on the proliferation of EC. (**D**) The effect of rotational speed on the proliferation of EC. Note: Uppercase letters indicate significant differences in the weight of EC between treatments, lowercase letters indicate significant differences in the sedimentation volume of EC between treatments, and treatments without letters indicate no significant difference. *p* < 0.05. Bar: SE error bars.

To enhance the proliferation efficiency of EC, a suspension culture was implemented. Maltose and sucrose were tested at various concentrations to determine their effects on callus growth. Results demonstrated that when maltose concentration was 10 g/L, the sedimentation volume of EC reached 16.25 mL, and its weight was 3.32 g, significantly higher than with other concentration treatments (*p* < 0.05) (Figure 2A). In contrast, no significant differences were observed across the six sucrose concentration gradients, with the amount of EC under the optimal sucrose treatment being lower than that achieved with maltose (*p* < 0.05) (Figure 2B). Consequently, 10 g/L maltose was selected as the optimal carbon source for the proliferation culture of *Pinus elliottii* EC.

After 7 days of suspension culture, the highest weight (2.43 g) and sedimentation volume (11.5 mL) of the EC were achieved at a pH of 5.8, significantly outperforming other treatments, indicating that this pH was most suitable for EC proliferation (*p* < 0.05) (Figure 2C). Additionally, no significant differences in EC growth were detected among agitation speeds of 120 rpm, 150 rpm, and 180 rpm; however, all three significantly outperformed the 60 rpm and 90 rpm treatments (*p* < 0.05) (Figure 2D). This phenomenon may be attributed to spatial constraints and nutrient diffusion limitations within the conical flasks.

### 2.3. Induction of Somatic Embryo Maturation in Pinus elliottii

(1) Pretreatment and Liquid–Solid Media Enhance Embryo Maturation Efficiency

**Figure 3:**
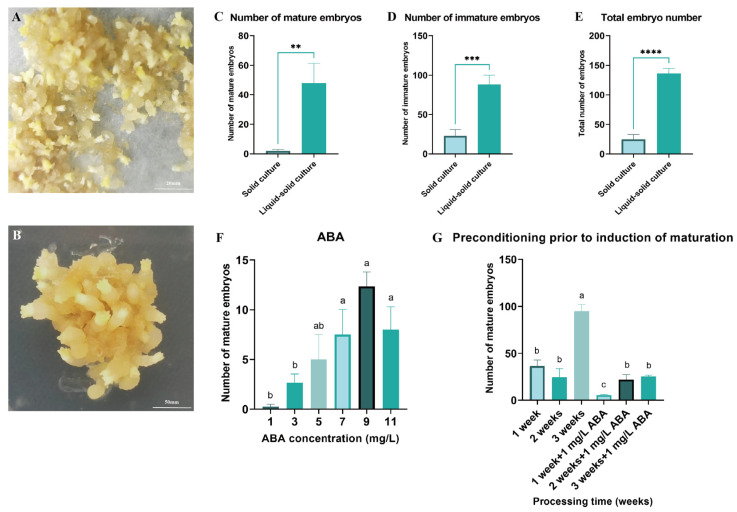
Induction of somatic embryo maturation in *Pinus elliottii*. (**A**) Liquid–solid induction of somatic embryo maturation in *Pinus elliottii*. (**B**) Solid-induced somatic embryo maturation in *Pinus elliottii*. (**C**) Statistical analysis of the number of mature somatic embryos. (**D**) Statistical analysis of the number of immature somatic embryos. (**E**) Overall statistics of the total embryo count. (**F**) Effect of ABA treatment on somatic embryo maturation. (**G**) Effect of pretreatment duration on somatic embryo maturation. Letters indicate significant differences among various treatments. *p* < 0.05, **: *p* < 0.01, ***: *p* < 0.001, ****: *p* < 0.0001. Bar: SE error bars.

Both solid and liquid–solid culture systems were utilized to induce somatic embryo maturation in *Pinus elliottii* (Figure 3A,B). The results demonstrated that the liquid–solid system significantly increased the yield of mature embryos, immature embryos, and total embryos compared to the solid system, with respective increases of 25.85-fold (mature), 3.86-fold (immature), and 5.51-fold (total) (*p* < 0.05) (Figure 3C–E). This enhancement is likely attributable to improved nutrient absorption facilitated by greater tissue–medium contact in the liquid–solid system.

Building upon this optimized maturation protocol, the optimal concentration of ABA and pretreatment duration were determined. The highest yield of mature embryos was achieved with 9 mg/L ABA and a 3-week pretreatment, significantly exceeding all other treatments (*p* < 0.05) (Figure 3F,G). Specifically, the number of mature embryos at an ABA concentration of 9 mg/L was 49.33 times greater than in the control group. After 3 weeks of pretreatment, an average of 94.75 mature embryos were induced per gram of EC, representing a substantial improvement in the somatic embryo maturation induction rate (*p* < 0.05).

(2) PSK and COS Boost Embryo Maturation Efficiency

**Figure 4:**
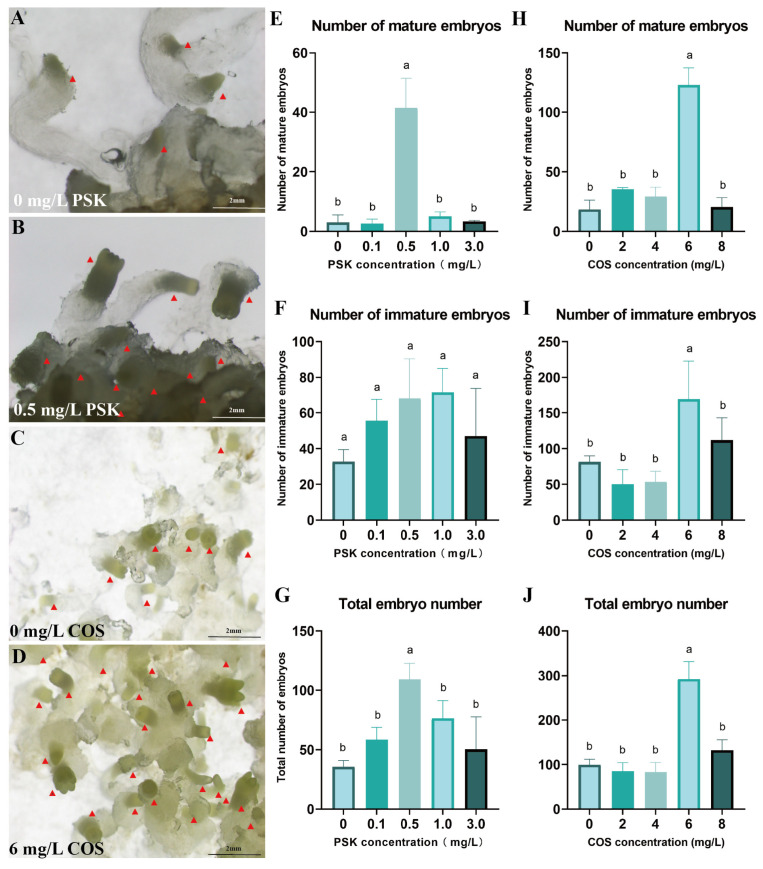
Optimization of conditions for somatic embryo maturation of *Pinus elliottii*. (**A**,**B**) PSK-induced mature somatic embryos; (**C**,**D**) COS-induced mature somatic embryos; (**E**–**G**) PSK-induced mature somatic embryos, immature somatic embryos, and total embryo count statistics; (**H**–**J**) COS-induced mature somatic embryos, immature somatic embryos, and total embryo count statistics. Letters indicate significant differences among various treatments. *p* < 0.05. Bar: SE error bars. Somatic embryos are marked with red triangles.

To improve the production of mature embryos, PSK was utilized for maturation induction. At 0.5 mg/L PSK, the yield reached 41.33 mature embryos and 109.33 total embryos, significantly exceeding all other concentration treatments (*p* < 0.05) (Figure 4E–G). Compared to the control, 0.5 mg/L PSK not only promoted embryonic development but also promoted the formation of well-differentiated embryonal heads (Figure 4A,B).

Furthermore, COS at 6 mg/L induced 292 total embryos with 123 mature embryos, significantly outperforming other concentrations (Figure 4C,D). This COS treatment resulted in 6.73-fold and 8.79-fold increases in mature embryo yield and total embryo production, respectively, relative to the control (*p* < 0.05) (Figure 4H–J).

(3) Activated Carbon Enhances Embryo Maturation Efficiency

**Figure 5:**
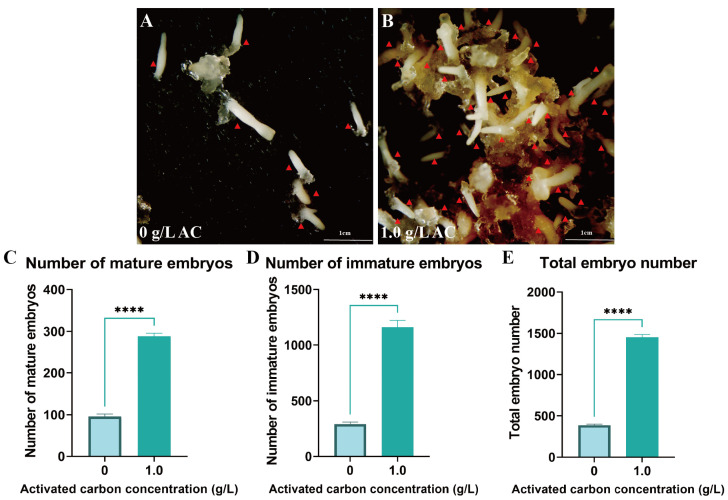
Addition of activated carbon promotes somatic embryo maturation in *Pinus elliottii*. (**A**,**B**) AC-induced somatic embryo maturation; (**C**–**E**) AC-induced mature somatic embryos, immature somatic embryos, and total embryo count statistics. *p* < 0.05, ****: *p* < 0.0001. Bar: SE error bars. Somatic embryos are marked with red triangles.

To further optimize embryogenesis efficiency, 1 g/L AC was supplemented to the medium. This treatment significantly improved somatic embryo maturation efficiency, inducing an average of 288.67 mature embryos per gram fresh weight of EC with a total embryo yield of 1452 (*p* < 0.05) (Figure 5A–E). Furthermore, AC supplementation significantly reduced the incidence of embryo browning through adsorption of phenolic compounds.

### 2.4. Somatic Embryo Germination and Seedling Transplantation of Pinus elliottii

**Figure 6:**
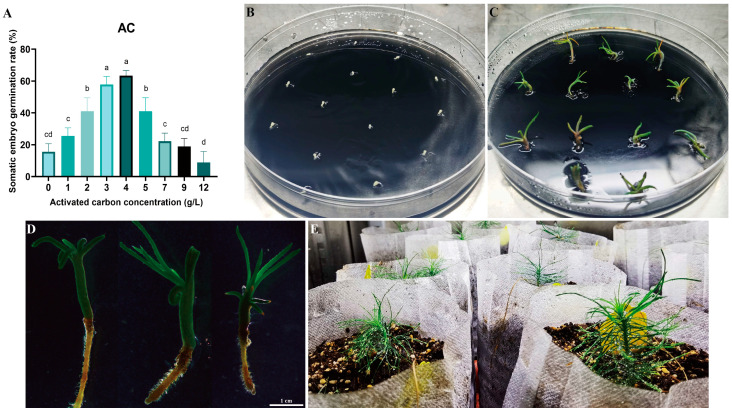
Somatic embryo germination and somatic embryo seedling transplantation of *Pinus elliottii*. (**A**) Effect of activated carbon concentration on somatic embryo germination; (**B**,**C**) induction of somatic embryo germination; (**D**,**E**) transplantation of somatic embryo seedlings to the greenhouse. Letters indicate significant differences among various treatments. *p* < 0.05. Bar: SE error bars.

**Figure 7:**
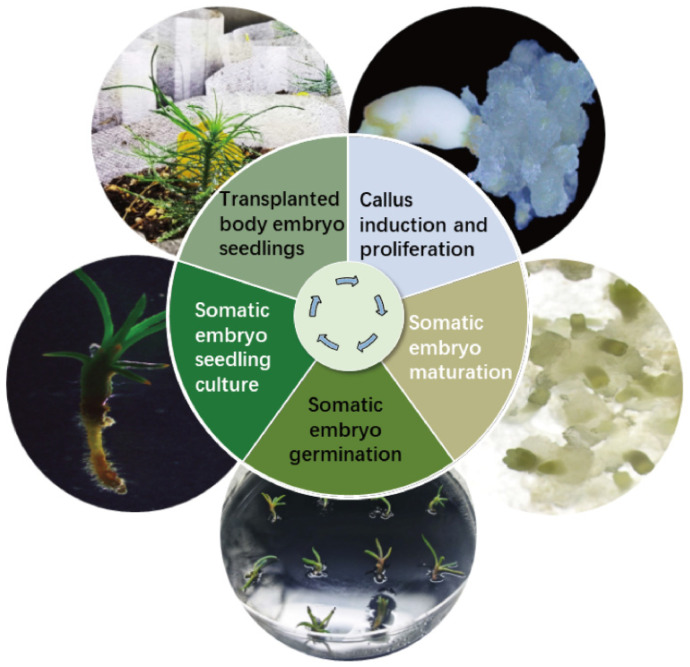
Schematic representation of the somatic embryogenesis technology system for *Pinus elliottii*.

Cotyledonary-stage somatic embryos derived from mature cultures were used to induce germination (Figure 6A–D). At an AC concentration of 4 g/L, the germination rate reached 63.00% (*p* < 0.05) (Figure 6A). Germinated somatic seedlings (Figure 6D) were placed in a light incubator for further seedling development. After 6–8 weeks, seedlings with vigorous growth and a well-developed root system were selected and transplanted into the substrate for greenhouse culture (Figure 6D,E). Survival rates were assessed 8 weeks post-transplantation, with 17 of 20 somatic seedlings surviving (85.00% survival rate). This protocol establishes a complete somatic embryogenesis system for *Pinus elliottii* (Figure 7).

## 3. Discussion

### 3.1. Genotypes Affect the Efficiency of Embryonic Callus Induction

The efficiency of EC induction is influenced by several factors, including plant species, explants, culture conditions, and plant growth regulators [31]. However, genetic factors are recognized as the primary determinants in somatic embryogenesis initiation [32]. In this study, immature zygotic embryos of *Pinus elliottii* were used as explants across 10 different genotypes. Using the same induction medium, we observed that the S2 genotype exhibited the highest number of EC inductions, while the V34 genotype showed the highest induction rate. Additionally, the induction of NEC in the V34, S2, and S16 genotypes was significantly lower than in the other seven genotypes. Similar genotype-related differences were observed in the induction of embryogenic callus in other pine species, such as *Pinus radiata* [33], *Pinus halepensis* [34], and hybrid *P. elliottii × P. caribaea* [35]. These findings suggest that genotypes with superior embryogenic callus induction should be further investigated to optimize culture conditions, providing valuable insights for future research on somatic embryogenesis.

### 3.2. Exogenous Phytohormones Affect Somatic Embryo Maturation

Plant growth regulators, particularly auxin and cytokinin, are essential for somatic embryogenesis induction [36]. Auxin acts as a pivotal regulator during early somatic embryogenesis [37]. However, the use of exogenous auxin often results in embryonic abnormalities. This occurs because auxin can trigger DNA methylation and mutations at the cellular level, which impede normal embryo development [38]. In contrast, hormone-free media can promote somatic embryo differentiation [39]. To mitigate the adverse effects of exogenous plant hormones on maturation, EC was pretreated for 3 weeks on LP basal medium devoid of hormones. This pretreatment significantly enhanced somatic embryo maturation, yielding 94.75 mature embryos per gram fresh weight of EC.

ABA plays a vital role in regulating cellular physiological metabolism and embryo development, particularly during the early stages of embryo and female gametophyte development [40]. Optimal ABA concentrations are well-established as key promoters of somatic embryo maturation [41,42]. Studies in *Paeonia ostii* further confirm ABA’s essential role in promoting embryonic morphogenesis and maturation [43]. In plant tissue culture, ABA is typically used to induce somatic embryos to enter the stationary phase [44]. For example, pretreatment for from 1 to 9 days in *Norway spruce* significantly increased the induction of mature embryos by over 10-fold [45]. In the study of somatic embryogenesis in *Quercus aliena*, it was revealed that the key genes *QaLEC2*, *QaCALS11*, and *QaSSRP1*, which are involved in this process, exhibited positive responsiveness to exogenous ABA [46]. In our study, 9 mg/L ABA increased mature embryo production in *Pinus elliottii* by 49.33-fold compared to ABA-free controls. These results suggest that the addition of ABA during somatic embryo maturation enhances the induction rate of cotyledon embryos, highlighting the crucial role of ABA in somatic embryo maturation.

### 3.3. PSK, COS Can Induce Somatic Embryo Maturation

PSK has been shown to enhance somatic embryogenesis in various plants, including *Cryptomeria japonica* [47]. The addition of 0.8 mg/L PSK increases the number of somatic embryos in *Liriodendron* hybrids, reduces the formation of malformed embryos, and improves plant regeneration efficiency [48]. Furthermore, 0.5 mg/L PSK promotes somatic embryogenesis progression in *Pinus massoniana*, facilitating the transition from pro-embryogenic mass (PEM) I to the PEMII or PEMIII stages of pro-embryos. It also supports the accumulation of soluble sugars, proteins, and starch during somatic embryo maturation [49]. In experiments involving *Pastinaca sativa* L. protoplasts for plant regeneration, it was observed that all regenerated plants originated from embryos developed in callus cultures on media supplemented with PSK [50]. PSK also stimulates protoplast division, callus induction, and somatic embryogenesis in *Angelica gigas Nakai* [51]. In research on Chinese fir somatic embryogenesis, Hao et al. found that PSK promotes somatic embryogenesis by regulating reactive oxygen species (ROS) levels, particularly reducing hydrogen peroxide (H_2_O_2_) accumulation [52]. PSK reduces ROS production by down-regulating peroxidase (PRX) expression, preventing ROS bursts, and ensuring successful embryonic suspensor transformation into somatic embryos. Moreover, PSK treatment increased the expression of *WOX2*, a key gene for somatic embryogenesis, indicating its regulatory effect on embryogenesis genes. In *Arabidopsis*, the heterologous expression of the *ClPSK* gene alleviated growth disorders caused by ROS bursts, further verifying PSK’s pivotal role in ROS regulation and supporting normal growth [52]. Several studies revealed that PSK significantly affected somatic embryo yield in resistant *Pinus elliottii* at a concentration of 1 mg/L, leading to a substantial increase in somatic embryo production [53]. In our study, at a concentration of 0.5 mg/L PSK, 41.33 mature embryos were obtained, with a total of 109.33 embryos. This treatment not only promoted the maturation of *Pinus elliottii* somatic embryos but also improved the structural integrity of the embryo head.

COSs are known to influence plant cell wall structure and signal transduction [54]. The mechanism of COS action involves regulating plant cell physiological responses through the activation of signaling molecules such as ROS and nitric oxide (NO) [54]. In *Nicotiana tabacum*, COS specifically binds to the cell wall and membrane, enhancing 1H-Indole-3-acetic acid (IAA) accumulation and reducing IAA peroxidase concentration in suspension cells, thereby promoting plant growth and development [55,56]. In *Liriodendron* hybrids, the addition of 0.05 mg/L COS significantly improved both the quantity and quality of somatic embryos, optimizing their developmental processes [57]. In our study, treatment with 6 mg/L COS induced 292 embryos, of which 123 were mature embryos. Compared to the control group, COS treatment resulted in a 6.73-fold increase in the number of mature embryos and an 8.79-fold increase in the total number of embryos.

### 3.4. Activated Carbon Can Induce Somatic Embryo Maturation and Germination

Activated carbon is a commonly used component in tissue culture media, primarily believed to absorb plant waste, toxic metabolites, and residual hormones [58]. Research has demonstrated that activated carbon can promote somatic embryo maturation [59,60]. In this study, we found that adding 1 g/L of activated carbon significantly improved the induction efficiency of cotyledon embryos in *Pinus elliottii*. On average, 288.67 mature embryos were induced per gram of embryogenic callus, and the total number of embryos reached 1452. The addition of activated carbon to the germination induction medium is a widely employed method for somatic embryogenesis in various conifers, such as *Pinus nigra* [61], *Pinus massoniana* [62], *Cryptomeria japonica* [63], and *Pinus armandii* [64]. Our study also showed that, at a concentration of 4 g/L, the germination rate of *Pinus elliottii* somatic embryos reached 63%. Similarly, the role of activated carbon in promoting somatic embryo maturation and germination has been reported in other conifers [65]. However, the precise mechanism by which activated carbon facilitates somatic embryo germination remains unclear. Jiang et al. proposed that activated carbon can absorb various components from the medium, including both beneficial and detrimental substances for somatic embryo germination [65]. Excessive hormones during somatic embryo maturation are a major barrier to germination. Theoretically, by absorbing the appropriate components, activated carbon can significantly enhance the germination rate of somatic embryos. Therefore, further investigation into the role of activated carbon and the determination of its optimal concentration for maximizing somatic embryo germination is essential.

## 4. Materials and Methods

### 4.1. Plant Materials

Immature seeds of *Pinus elliottii* were collected from two genetically distinct sources: high-resin-yielding genotypes from the resin seed orchard at Changle Forest Farm in Yuhang, Zhejiang Province (30°20′13.517″ N, 119°51′13.054″ E), and fast-growing genotypes from the first-generation clonal seed orchard in Xiajiang County, Ji’an City, Jiangxi Province (27°34′29.291″ N, 115°21′51.682″ E). Ten open-pollinated mother trees were selected, including high-yield resin trees V5, V10, V11, V26, V30, V33, V34) and fast-growing trees (S1, S2, S16). The cones were collected and stored at 4 °C for later processing. Following cone removal, fully developed seeds were selected and transferred to an ultra-clean bench for disinfection. The seeds were first disinfected with 75% alcohol for 30 s, followed by three rinses in sterile water. They were then treated with 10% sodium hypochlorite for 10 min and rinsed five times, with continuous agitation to ensure thorough contact. After drying with sterile filter paper, the outer and inner integuments were peeled off to obtain sterile immature embryos.

### 4.2. Culture Conditions

The H1 medium was developed by modifying the basal LP medium [66], supplemented with additional components: 30 g/L maltose (Sangon Biotech, Shanghai, China), 450 mg/L L-glutamine (Merck, Darmstadt, Germany), 500 mg/L casein hydrolysate (Merck), 500 mg/L myo-inositol (Merck), and 250 mg/L 2-(N-morpholino)ethanesulfonic acid monohydrate (MES; Sangon Biotech, Shanghai). Prior to autoclave sterilization at 121 °C for 20 min, the pH was adjusted to 5.8 with 4 g/L gellan gum (Sangon Biotech, Shanghai) incorporated as a solidifying agent. Each experimental group included ≥3 biological replicates. Post-inoculation cultures were maintained at 23 °C ± 1 °C under continuous darkness, with morphological observations recorded at regular intervals.

### 4.3. Callus Induction Study

Immature zygotic embryos were cultured on H1 medium supplemented with 2.0 mg/L 2,4-dichlorophenoxyacetic acid (2,4-D) and 2.5 mg/L kinetin (KT) for callus induction. Each dish contained 10 explants with ≥3 biological replicates per treatment. Cultures were maintained at 23 °C ± 1 °C under continuous darkness for 4 weeks prior to evaluation. Quantitative assessment included: (1) total explant count, (2) number of embryogenic callus (EC) formations, (3) number of non-embryogenic callus (NEC) formations, with induction rates calculated as (EC count/total explants) × 100% and (NEC count/total explants) × 100% respectively.

### 4.4. Optimization of Suspension Culture Conditions for Embryogenic Callus

EC of the S2 genotype—selected for its stable proliferation traits—was used to establish suspension cultures in 150 mL conical flasks containing 30 mL liquid medium inoculated with 1 g fresh weight EC. A single-factor experimental design tested four parameters: maltose concentration (0, 5, 10, 15, 20, 30 g/L); sucrose concentration (0, 5, 10, 15, 20, 30 g/L); pH (4.9, 5.2, 5.5, 5.8, 6.1, 6.4, 6.7); and agitation speed (60, 90, 120, 150, 180 rpm). Each treatment comprised three biological replicates cultured in darkness at 23 °C ± 1 °C for 7 days. Proliferation was assessed using sedimentation cell volume (SCV) and fresh weight. SCV was determined by measuring the volume of precipitated callus using a graduated cylinder, while fresh weight was obtained by filtering the callus to remove excess water and subtracting the weight of the filter paper.

### 4.5. Optimization of Maturation Induction Conditions for Pinus elliottii

EC of the V30 genotype—selected for stable proliferation and morphological consistency—was subjected to somatic embryo maturation induction. After 60 days, mature embryo counts were quantified based on morphological criteria: cotyledonary-stage embryos exhibiting ≥4 radially arranged cotyledons, smooth hypocotyls, and distinct radicles were classified as mature; all other developmental stages were designated immature. One gram of embryogenic callus was divided into 10 pieces, which were placed on a solid induction medium containing gellan gum. Alternatively, the callus was mixed with 5 mL of a liquid induction medium (without gellan gum), spread onto filter paper, and then transferred to a solid medium. Tested supplements included: abscisic acid (ABA; Merck) at 1–11 mg/L (6 gradients), phytosulfokine (PSK; NovoPro, Shanghai, China) at 0–3.0 mg/L (5 gradients), chitooligosaccharide (COS; Maokang, Shanghai, China) at 0–8 mg/L (5 gradients), and activated carbon (AC; Merck) at 0–1 g/L. To evaluate the impact of pre-culture on maturation induction, the embryogenic callus was initially placed on medium devoid of exogenous plant hormones, followed by maturation induction after 1, 2, or 3 weeks of pre-culture. Each treatment was repeated at least three times and cultured in continuous darkness at 23 °C ± 1 °C.

### 4.6. Somatic Embryo Germination and Transplantation

Mature somatic embryos were transferred to H1 medium to induce germination. A single-factor design assessed AC concentrations (0, 1, 2, 3, 4, 5, 7, 9, 12 mg/L) on germination efficiency. Germination was initially conducted under dark conditions for 7 days, after which the cultures were transferred to light conditions. The light conditions consisted of a light intensity of 120 μmol·m^2^·s^−1^, a photoperiod of 16 h/8 h. Germination rates were recorded after 30 days. After germination, the somatic embryo seedlings were maintained in sterile bottles for 4–6 weeks. Following one week of acclimatization at room temperature, the seedlings were transferred to a growth medium (peat soil:perlite = 3:1) and placed in a light incubator (16 h/8 h light period, light intensity of 120 μmol·m^2^·s^−1^, temperature 23 °C ± 1 °C, humidity 90%). Survival rates were determined after 40 days based on functional root development and new needle emergence.

### 4.7. Data Statistics and Analysis

Data are presented as mean ± standard deviation. All analyses were performed using SPSS 26.0 (IBM, Chicago, IL, USA), with graphical representations generated in GraphPad Prism 9.0 (GraphPad Software, Santiago, MN, USA). Prior to parametric testing, normality was verified by the Shapiro–Wilk test (*p* > 0.05) and residual histograms, while homogeneity of variances was assessed via Levene’s test (*p* > 0.05). When variances were unequal (Levene’s *p* ≤ 0.05), Brown–Forsythe correction and Welch ANOVA were applied. For data meeting both assumptions, one-way ANOVA followed by Duncan’s multiple comparison test (for homogeneous variances) or Games–Howell test (for heterogeneous variances) was used to determine inter-group differences. Statistical significance was defined at α = 0.05.

## 5. Conclusions

This study focused on optimizing and applying somatic embryogenesis technology for *Pinus elliottii*, aiming to identify key conditions for the induction of somatic embryo maturation. The results revealed that the induction of embryogenic callus in *Pinus elliottii* was significantly influenced by genotype. The liquid–solid induction method significantly increased the number of mature embryos, yielding 25.85 times more than the conventional method. Further analysis revealed that a 3-week pretreatment, combined with specific concentrations of ABA, PSK, and COS (ABA 9 mg/L, PSK 0.5 mg/L, COS 6 mg/L), significantly improved somatic embryo maturation efficiency. Additionally, the inclusion of activated carbon was crucial for both the maturation and germination of somatic embryos. During the induction of somatic embryo maturation, 1 g/L of activated carbon induced 288.67 mature embryos per gram of embryogenic callus, with a total of 1452 embryos. In the germination phase, applying 4 g/L of activated carbon resulted in a somatic embryo germination rate of 63%, and the survival rate of somatic embryo-derived seedlings reached 85%. In conclusion, this study established an efficient method for inducing somatic embryo maturation in *Pinus elliottii*.

## Data Availability

Data is contained within the article.

## Abbreviations

ABA	abscisic acid
PEG	polyethylene glycol
NH4^+^	ammonia
NO3^−^	nitrate
2,4-D	2,4-dichlorophenoxyacetic acid
BA	6-benzylaminopurine
KT	kinetin
PSK	Phytosulfokine
COS	Chitooligosaccharide
AC	Activated Charcoal
EC	embryogenic callus
NEC	non-embryogenic callus
ROS	reactive oxygen species
H_2_O_2_	hydrogen peroxide
PRX	peroxidase
NO	nitric oxide
IAA	1H-Indole-3-acetic acid
